# The impact of workplace psychosocial factors on menstrual disorders and infertility: a protocol for a systematic review and meta-analysis

**DOI:** 10.1186/s13643-022-02066-4

**Published:** 2022-09-07

**Authors:** Natsu Sasaki, Kotaro Imamura, Kazuhiro Watanabe, Yui Hidaka, Emiko Ando, Hisashi Eguchi, Akiomi Inoue, Kanami Tsuno, Yu Komase, Mako Iida, Yasumasa Otsuka, Asuka Sakuraya, Yumi Asai, Mai Iwanaga, Yuka Kobayashi, Reiko Inoue, Akihito Shimazu, Akizumi Tsutsumi, Norito Kawakami

**Affiliations:** 1 Department of Mental Health, Graduate School of Medicine, The University of Tokyo, Tokyo, Japan; 2 Department of Digital Mental Health, Graduate School of Medicine, The University of Tokyo, 7-3-1, Hongo, Bunkyo-ku, Tokyo, 113-0033 Japan; 3 Department of Public Health, Kitasato University School of Medicine, Sagamihara, Japan; 4 Institute for Cancer Control, National Cancer Center, Tokyo, Japan; 5 Department of Mental Health, Institute of Industrial Ecological Sciences, University of Occupational and Environmental Health, Kitakyushu, Japan; 6 Institutional Research Center, University of Occupational and Environmental Health, Kitakyushu, Japan; 7 School of Health Innovation, Kanagawa University of Human Services, Kawasaki, Japan; 8 Department of Psychiatric Nursing, Graduate School of Medicine, The University of Tokyo, Tokyo, Japan; 9 Faculty of Human Sciences, University of Tsukuba, Tokyo, Japan; 10 Faculty of Policy Management, Keio University, Tokyo, Japan; 11 Faculty of Social Policy & Administration, Hosei University, Tokyo, Japan

**Keywords:** Biopsychosocial medicine, Endocrine, Gynecology, Occupational health, Reproductive health

## Abstract

**Introduction:**

Workplace environment, especially psychosocial factors at work such as job strain, workplace social support, and shift work, may affect the menstrual abnormalities and fertility of female workers. However, the association between psychosocial factors at work and menstrual abnormalities or fertility is not well understood. To address this relationship, we will conduct a systematic review and a meta-analysis of the literature that has utilized a longitudinal or prospective cohort design.

**Methods and analysis:**

The inclusion criteria for this systematic review and meta-analysis are defined as follows: (P) adult female workers (over 18 years old), (E) the presence of adverse psychosocial factors at work, (C) the absence of adverse psychosocial factors at work, and (O) any menstrual cycle disorders, menstrual-related symptoms, or fertility. The MEDLINE, Embase, PsycINFO, PsycArticles, and Japan Medical Abstracts Society electronic databases will be used to search for published studies. The statistical synthesis of the studies included in the meta-analysis will be conducted to estimate pooled coefficients and 95% CIs. For the main analysis, we will synthesize measures of association between psychosocial factors at work and menstrual-related disorders/symptoms. At least three eligible studies will have to be gathered to conduct a meta-analysis; otherwise (i.e., if only one or two studies will be eligible and included), the results will be presented in a narrative table. We will use the Risk of Bias in Non-randomized Studies of Interventions (ROBINS-I) to determine the quality of selected studies. To assess meta-bias, Egger’s test, along with a funnel plot, will be used to check for publication bias. Lastly, we will examine heterogeneity using the *χ*^2^ test with Cochran’s Q statistic and *I*^2^.

**Ethics and dissemination:**

The results and findings will be submitted and published in a scientific peer-reviewed journal and will be disseminated broadly to researchers and policymakers interested in the translatability of scientific evidence into good practices.

**Systematic review registration:**

The study protocol was registered at the UMIN registry (registration number: UMIN000039488). The registration date is on 14 Feb 2020.

URL: https://upload.umin.ac.jp/cgi-bin/ctr/ctr_view_reg.cgi?recptno=R000044704

**Supplementary Information:**

The online version contains supplementary material available at 10.1186/s13643-022-02066-4.

## Strength and limitations of this study

• This systematic review and a meta-analysis will investigate the associations between psychosocial factors at work and menstrual abnormalities or fertility.

• This review will include only the literature that has utilized a longitudinal design.

• Various outcomes related to reproductive health in female workers will be examined (e.g., fertility, premenstrual symptoms, cycle disorders, menopausal symptoms).

**Supplementary Information:**

The online version contains supplementary material available at 10.1186/s13643-022-02066-4.

## Introduction

The female working participation rate has been increasing along with improved educational opportunities and the lack of labor force [1]. Poor reproductive health of working women, including infertility and menstrual abnormalities, has a major effect on health and work outcomes throughout the preconception period (before pregnancy) to the climacteric period [2]. Reproductive health among female workers can be a social issue as well as affect females’ career choices.

Menstrual abnormalities can be differentiated into menstrual cycle disorders and associated menstrual symptoms. Menstrual cycle disorders, including shorter or longer cycles in reproductive age, are related to infertility [3–5], breast and ovarian cancer [6, 7], diabetes [8], and cardiovascular disease [9, 10]. In addition to age, weight, tumor, inflammation, or endocrine dysfunctions, psychosocial factors can affect the menstrual cycle. In general, the menstrual cycle is regulated by hypothalamic gonadotropin-releasing hormone (GnRH); however, the function and secretion of GnRH can be inhibited by hormones released by the hypothalamic-pituitary-adrenal (HPA) axis, which is activated in response to stressors [11, 12]. The stress reactions can directly change the level of serum concentrations of sex hormones in response to psychosocial factors [13]. It may affect fertility outcomes, such as contraception or pregnancy. Female workers facing various types of stressors may be at risk of menstrual cycle disorders and infertility.

Alongside the menstrual cycle disorders and adverse fertility outcomes, menstrual-related symptoms (e.g., pelvic pain, premenstrual syndrome [PMS], menopausal symptoms) have a considerable effect on workers’ quality of life (QOL) and diminished work capacity [14–16]. The cost of productivity loss was reported US $15,737 per working woman per year due to absenteeism and presenteeism for endometriosis alone, which is a major cause of heavy pelvic pain [17]. A longitudinally designed study revealed that PMS among female workers was associated with higher absenteeism and less work productivity [18]. Furthermore, the high prevalence of menstrual-related symptoms has been reported worldwide, in particular chronic pelvic pain (CPP): 24% [19], PMS: 30–40%, and premenstrual dysphoric disorder (PMDD) (a more serious variant of PMS) 3–8% [20].

Cytokines/chemokines and muscle contractions are a direct cause of menstrual pain [21]. Still, psychosocial factors at work, such as shift work, low support, or high demand, may play a role in alleviating or aggravating this pain. Stress reactions can also strengthen PMS through difficulty in mood regulation and increasing sensitivity to stressful experiences [22, 23]. Regarding vasomotor symptoms in perimenopause (e.g., hot flushes), psychosocial factors can be in charge of exacerbating symptoms [24]. Therefore, it is important to identify psychosocial factors (i.e., stressors) at work that affect menstrual-related symptoms and explore the mechanisms through which they exert this effect.

Occupational psychosocial environments thus have a non-neglectable effect on female menstrual health. Previous literature reviews have revealed some physical and chemical workplace factors (e.g., chemical exposure, low temperature at work, lifting heavyweight) related to menstrual cycle disorders, dysmenorrhea, and reproductive outcome [25, 26]. However, limited evidence has supported the relationship between psychosocial factors at work and menstrual outcomes. Accumulating evidence indicates the adverse health effects of shift work. The meta-analysis of Stocker et al. showed that shift work increased the risk of menstrual disruption and infertility [27]. Shift work can contribute to cycle disorder by changing the gonadotropin secretion cycle as a result of circadian rhythm disturbances as well as stress-related dysfunction of the hypothalamus-pituitary-ovarian axis [28, 29]. Concerning menstrual-related symptoms, a cross-sectional survey in the workplace revealed that low job control, low co-worker support, and low job security were found to be associated with a higher risk for menstrual pain [30]. Somatic symptoms in perimenopause are also known to be affected by work-related psychosocial factors, such as supervisor support [31].

However, psychosocial factors in the workplace associated with menstrual abnormalities in female workers have not been comprehensively investigated. Moreover, no meta-analysis with well-designed longitudinal studies has been conducted to our knowledge.

### Objectives

This systematic review and a meta-analysis study aims to investigate the comprehensive association between psychosocial factors at work and menstrual-related disorders/symptoms using longitudinal data.

## Methods and analysis

### Study design

**Fig. 1:**
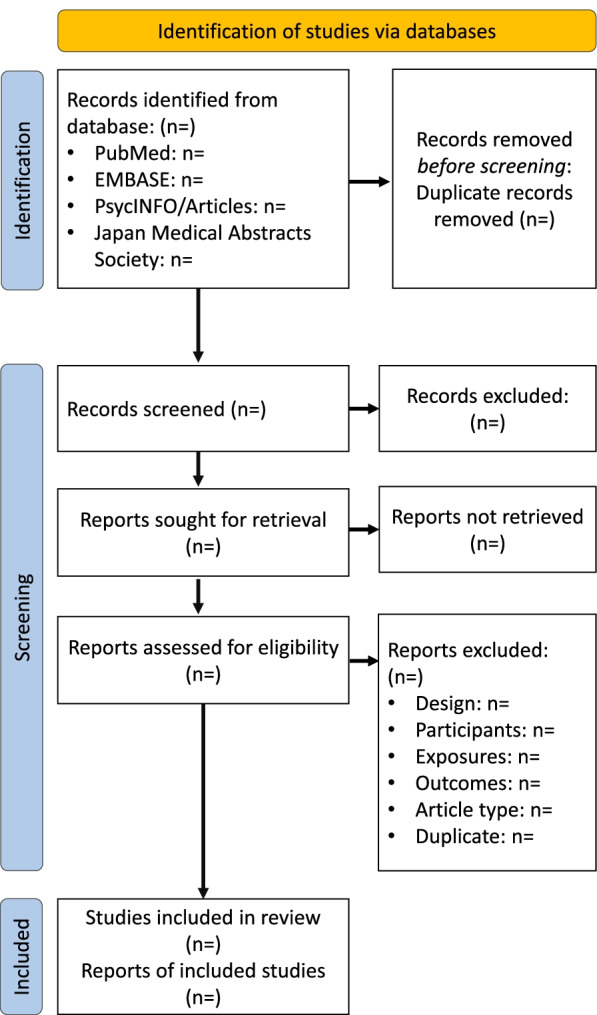
PRISMA 2020 flow diagram of systematic review search results

The protocol is being reported in accordance with the Preferred Reporting Items for Systematic Review and Meta-Analysis Protocols (PRISMA-P) statement [32]. The study protocol was registered at the UMIN registry (registration number: UMIN000039488). The registration date is on 14 Feb 2018. Figure 1 summarizes the flow of the systematic review process. The PRISMA-P checklist can be available in Supplemental appendix 1.

### PECO and eligibility criteria

For this systematic review and meta-analysis, the eligible participants, exposures, comparisons, and outcomes (PECO) of the studies will include the following:

- (P) Adult female workers (over 18 years old)
- (E) The presence of adverse psychosocial factors at work
- (C) Absence of adverse psychosocial factors at work
- (O) Any menstrual cycle disorders, menstrual-related symptoms, or fertility

Job strain, effort-reward imbalance, working hours, shift work, low social support, and other organizational-level factors, work conditions, and interpersonal relationships at work will be included as adverse psychosocial factors.

Menstrual disorders and related symptoms refer to diverse menstrual dysfunctions (e.g., cycle disorder, hypermenorrhea), gynecological disease/syndrome (e.g., endometriosis, polycystic ovarian syndrome), menstrual-related symptoms (e.g., PMS, menopausal symptoms), reproductive outcomes (e.g., infertility, time to be pregnant), and biological outcomes (e.g., serum sex hormone).

Inclusion criteria are as follows:

1. Studies that included female participants who were working as of baseline survey period
2. Studies which assessed the adverse psychosocial factors at work as exposure variables at baseline survey.
3. Studies that assessed any menstrual-related or fertility-related outcome at baseline and follow-up surveys
4. Studies which used a longitudinal or prospective cohort design
5. Studies are written in English or Japanese
6. Studies which have been published by peer-reviewed journal (including advanced online publication)

Exclusion criteria are as follows:

1. Studies targeting pregnancy-related outcomes (e.g., premature birth)
2. Studies targeting malignant outcomes (e.g., cancer)

### Information sources and search strategy

A preliminary search of PROSPERO, MEDLINE, and the Cochrane Database of Systematic Reviews was conducted to identify if there is any systematic review protocol on the topic and did not find any systematic review protocols with the same design. The MEDLINE, Embase, PsycINFO, PsycArticles, and Japan Medical Abstracts Society electronic databases will be used to search for published studies. The search terms will include words related to the PECO of the studies (Supplementary Appendix 2). The search terms for psychosocial factors at work will be referred the previous our meta-analysis [33–35]. The complete search term was developed by critical periluminal review of relevant articles and indexing terms, including a range of task and organizational characteristics and work conditions, such as job strain, social support, effort-reward imbalance, organizational injustice, workplace social capital, long working hours, and shift work. The search terms for outcome variables will be carefully selected by reviewing current evidence regarding menstrual-related health. The authors consulted a public health medical doctor specializing in obstetrics and gynecology (outside the research team) to confirm the search terms. Infectious or inflammatory diseases and pelvic organ prolapse were not included in outcomes. Terms of menstrual-related outcomes were retrieved from previous systematic reviews [17, 27, 36–38].

### Study records

Microsoft Excel (Washington, USA) will be used to manage the data. Before screening the studies, NS will remove duplicate entries from the Excel file.

### Selection process

Selection process will be shared to our team members, who are all with extensive experiences in systematic review to make the screening time short. In addition, critical discussion in eligibility criteria will contribute to increase the accuracy of screening. Nineteen investigators (NS, KI, KW, EM, HE, AI, KT, YH, YKom, MIi, YO, ASa, YA, MIw, YKob, RI, ASh, AT, and NK) will screed the studies independently according to the eligibility criteria. After excluding duplicate records, the remaining articles will be distributed to 16 investigators (NS, KI, KW, EM, HE, AI, KT, YH, YKom, MIi, YO, ASa, YA, MIw, YKob, and RI). Two investigators will receive the same set of articles. Each investigator will screen the title and abstract of each article independently to select eligible studies according to the eligibility criteria (sifting phase). In this phase, we will gather the full texts of all eligible studies. Subsequently, two investigators will review the full texts independently. Any disagreements will be settled by consensus among all authors, and the reasons for excluding studies will be documented.

### Data collection process

Data will be extracted from the included studies independently by 16 investigators (NS, KI, KW, EM, HE, AI, KT, YH, YKom, MIi, YO, ASa, YA, MIw, YKob, and RI) using a standardized data extraction form. The authors will discuss any disagreements or inconsistencies until a consensus is achieved. From the included articles, the investigators will extract information on publication year, study design, the country of study origin, the number of participants completing the baseline survey and included in the statistical analysis, demographic characteristics of the participants (i.e., age, occupation), the length of follow-up and attrition rate, exposure variables (i.e., adverse psychosocial factors at work), outcome variables (i.e., menstrual abnormalities, fertility), and data necessary to calculate the coefficients (β, γ), odds ratios (ORs), relative risks (RRs) or hazard ratios (HRs) with standard errors (SEs), or 95% confidence intervals (CIs) to determine the association between psychosocial factors at work and menstrual abnormalities or fertility. In case of missing data, we will try to contact the authors of the included studies to obtain missing information.

### Synthesis methods

The outcomes of included studies will be integrated in a meta-analysis and stratified by types of measures of association (β, γ, OR, RR, and HR). Subsequently, pooled coefficients and 95% CIs will be estimated. For the studies that reported ORs, RRs, or HRs, we will calculate log-transformed ORs, RRs, or HRs and determine SEs based on 95% CIs. Psychosocial factors at work variables (types of exposure) will be categorized according to some specific work-related stress models (e.g., job demand-control (or job strain)/demand-control-support model, effort-reward imbalance model). Outcome variables will be classified by considering the concepts that the included study specifies it measures and by referring to the existing studies and models in menstrual disorders and fertility. A funnel plot and Egger’s test will be used to plot these parameters and to examine publication bias.

#### Primary analyses

For the main analysis, we will synthesize all types of psychosocial factors at work and all types of menstrual-related disorders/symptoms. The outcomes assessed on a continuous scale will be converted to dichotomous variables based on reasonable and theoretically sound cutoff points. Dichotomous and continuous variables for which no reasonable cutoff point could be determined will be analyzed separately.

At least three eligible studies will have to be gathered to conduct a meta-analysis; otherwise (i.e., if only one or two studies will be eligible and included), the results will be presented in a narrative table. All the studies included will be presented in the table, independent of the methodological quality result. A fixed-effect model will be used with homogeneous data; otherwise, a random-effects model will be used. The chi-square test with Cochran’s Q statistic and *I*^2^ will be used to test heterogeneity. *I*^2^ values of 25%, 50%, and 75% indicate low, medium, and high heterogeneity, respectively.

### Subgroup and sensitivity analyses

Subgroup analyses will be conducted to compare the results under a specific type of exposure (e.g., night shift, high job strain) and outcome variables (e.g., menstrual cycle disorder, PMS), if we have enough data to conduct such analyses. Meta-regression will be conducted in case of significant pooled associations with any grouping characteristics. A sensitivity analysis will be run for studies in which the Risk of Bias in Non-Randomized Studies of Interventions (ROBINS-I) is classified as low risk [39].

### Risk of bias in individual studies and assessment of meta-bias

The 16 investigators (NS, KI, KW, EM, HE, AI, KT, YH, YKom, MIi, YO, ASa, YA, MIw, YKob, and RI) will utilize the internationally recognized tool for evaluating the risk of bias (ROBINS-I) to independently assess the quality of included studies [39]. The risk of bias will be classified as low, high, or unclear. Any discrepancies between the investigators in the quality assessment will be documented and discussed until achieving a consensus. A summary of findings (SoF) will be created using the Grading of Recommendations, Assessment, Development and Evaluation (GRADE) approach to grade the certainty of evidence.

### Patient and public involvement

There is no direct patient or public involvement in the design of this study.

## Ethics and dissemination

Since our data will be extracted from published studies, and thus privacy issues will not be of concern, ethical approval will not be needed to apply this review protocol. Results and the findings will be submitted to a scientific peer-reviewed journal for publication and disseminated broadly to researchers and policymakers interested in the translatability of scientific evidence into good practices.

### Strengths and limitations

This will be the first systematic review and meta-analysis to explore the comprehensive effects of a wide range of psychosocial factors at work on menstrual-related disorders among female workers. The findings of this study will establish the link between psychosocial factors at work and menstrual abnormalities or fertility by restricting the eligible studies to those that utilized longitudinal or prospective cohort design. Thus, reviewing the current evidence would contribute to promoting women’s health at the workplace throughout preconception to menopause. Moreover, it would guide the development and implementation of strategies to improve psychosocial factors at work that affect working women.

Nevertheless, this systematic review and meta-analysis study is not without limitations. The characteristics of the participants included in the selected studies may limit the generalization of the findings. The database which will be used in this review is selected based on the previous research, but not all databases are exhaustive.

## Supplementary Information

**Additional file 1.** PRISMA-P 2015 Checklist.

**Additional file 2.** Search terms for PubMed.

## Data Availability

Not applicable.
