# Supplementary material for: The impact of workplace psychosocial factors on menstrual disorders and infertility: a protocol for a systematic review and meta-analysis
Source: Syst Rev. 2022 Sep 7;11:195. doi: 10.1186/s13643-022-02066-4 (PMC9581335; doi:10.1186/s13643-022-02066-4)
Supplement: Supplementary file 2 — Additional file 2. Search terms for PubMed. [file 13643_2022_2066_MOESM2_ESM.docx]

**Supplementary Appendix 2**

Search terms for PubMed

(employe*[tw] OR manag*[tw] OR colleague*[tw] OR worksit*[tw] OR “work”[tw] OR works*[tw] OR work’*[tw] OR worka*[tw] OR worke*[tw] OR workg*[tw] OR worki*[tw] OR workl*[tw] OR workp*[tw] OR occupant*[tw] OR company*[tw] OR offic*[tw] OR busines*[tw] OR workplace[mh]) AND (("Stress, Mechanical"[Mesh] OR "Lifting"[Mesh] OR "Moving and Lifting Patients"[Mesh] OR "Weight-Bearing"[Mesh] OR "Biomechanics" OR “Physical Exertion"[Mesh] OR "Torsion, Mechanical"[Mesh] OR "Postural Balance"[Mesh] OR "Walking"[Mesh] OR "Recovery of Function"[Mesh] OR "Relaxation"[Mesh] OR (static[Title/Abstract] AND posture) OR (awkward[Title/Abstract] AND posture) OR (dynamic[Title/Abstract] AND posture) OR static work[Title/Abstract] OR dynamic load*[Title/Abstract] OR lift*[Title/Abstract] OR carry*[Title/Abstract] OR hold*[Title/Abstract] OR pull*[Title/Abstract] OR drag*[Title/Abstract] OR push*[Title/Abstract] OR manual handling[Title/Abstract] OR force*[Title/Abstract] OR biomechanic*[Title/Abstract] OR walking*[Title/Abstract] OR postural balance[Title/Abstract] OR flexion*[Title/Abstract] OR extension*[Title/Abstract] OR turning[Title/Abstract] OR sitting[Title/Abstract] OR kneeling[Title/Abstract] OR squatting[Title/Abstract] OR twisting[Title/Abstract] OR bending[Title/Abstract] OR reaching[Title/Abstract] OR standing[Title/Abstract] OR sedentary[Title/Abstract] OR repetitive movement*[Title/Abstract] OR monotonous work[Title/Abstract] OR relaxation[Title/Abstract] OR recovery of function[Title/Abstract] OR physical demand*[Title/Abstract] OR physically demand*[Title/Abstract]) OR ("Stress, Psychological"[Majr] OR "Social Support"[Majr] OR "Job Satisfaction"[Mesh] OR "Work Schedule Tolerance"[Mesh] OR "Employee Performance Appraisal"[Mesh] OR "Employee Grievances"[Mesh] OR "Social Justice/psychology"[Mesh] OR "Personnel Downsizing"[Mesh] OR "Staff Development"[Mesh] OR "Organizational Culture"[Mesh] OR "Bullying"[Mesh] OR "Prejudice"[Mesh] OR "Social Discrimination"[Mesh] OR "Interpersonal Relations"[Mesh] OR "Communication/psychology"[Mesh]) OR (psychosocial[Title/Abstract] OR job strain[Title/Abstract] OR work strain[Title/Abstract] OR work demand*[Title/Abstract] OR job demand*[Title/Abstract] OR high demand*[Title/Abstract] OR low control[Title/Abstract] OR lack of control[Title/Abstract] OR work control[Title/Abstract] OR job control[Title/Abstract] OR decision latitude[Title/Abstract] OR work influence*[Title/Abstract] OR demand resource*[Title/Abstract] OR effort reward*[Title/Abstract] OR time pressure*[Title/Abstract] OR recuperation*[Title/Abstract] OR work overload*[Title/Abstract] OR work over-load*[Title/Abstract] OR recovery[Title/Abstract] OR coping[Title/Abstract] OR work ability[Title/Abstract] OR social support[Title/Abstract] OR support system*[Title/Abstract] OR social network*[Title/Abstract] OR emotional support[Title/Abstract] OR interpersonal relation*[Title/Abstract] OR interaction*[Title/Abstract] OR social capital [Title/Abstract] OR justice*[Title/Abstract] OR injustice*[Title/Abstract] OR job satisfaction[Title/Abstract] OR work satisfaction[Title/Abstract] OR boredom[Title/Abstract] OR skill discretion*[Title/Abstract] OR staff development[Title/Abstract] OR discrimination[Title/Abstract] OR harass*[Title/Abstract] OR work-place conflict*[Title/Abstract] OR workplace violen*[Title/Abstract] OR work-place violen*[Title/Abstract] OR bullying[Title/Abstract] OR ageism[Title/Abstract] OR homophobia[Title/Abstract] OR racism[Title/Abstract] OR sexism[Title/Abstract] OR victimization*[Title/Abstract] OR silent workplace*[Title/Abstract] OR role ambiguity[Title/Abstract] OR role-conflict*[Title/Abstract] OR work-role*[Title/Abstract] OR working hour*[Title/Abstract] OR working time[Title/Abstract] OR day-time[Title/Abstract] OR night-time[Title/Abstract] OR shift work*[Title/Abstract] OR work shift*[Title/Abstract] OR temporary work[Title/Abstract] OR full-time[Title/Abstract] OR part-time[Title/Abstract] OR flexible work*[Title/Abstract] OR organizational change[Title/Abstract] OR organisational change[Title/Abstract] OR lean production[Title/Abstract] OR job security[Title/Abstract] OR job insecurity[Title/Abstract])) AND (menstruation OR menstrual OR “menstrual pain” OR “period pain” OR “period pains” OR menorrhagia OR hypermenorrhea OR dysmenorrhea[MeSH] OR premenstrual OR “PMS” OR “premenstrual syndrome” OR “PMDD” OR “premenstrual dysphoric disorder” OR amenorrhea OR menoxenia OR metrorrhagia OR endometrio* OR myoma* OR leiomyoma OR fibroids OR adenomyosis OR ovarian cyst* OR endometrioma OR polycystic ovar* OR menopaus* OR hot flush* OR climacteric OR estrogen* OR estradiol OR progesterone* OR prolactin OR lutenizing OR LH OR “lutenizing hormone” OR follicul* OR FSH OR “follicle stimulating hormone” OR gonadotropin OR GnRH OR “gonadotropin releasing hormone” OR “basal body temperature” OR infertility OR fertility OR fecund* OR subfecundity OR reproductiv*) AND (longitudinal OR prospective OR cohort OR (follow AND up) OR observational OR (case AND (control or cohort)) OR case-control OR case-cohort)
